# Design, Analysis, and Simulation of a MEMS Tuning Fork Gyroscope with a Mechanical Amplification Structure

**DOI:** 10.3390/mi16020195

**Published:** 2025-02-08

**Authors:** Haotian Hu, Benedetta Calusi, Alvise Bagolini, Maria F. Pantano

**Affiliations:** 1 Department of Civil, Environmental and Mechanical Engineering, University of Trento, Via Mesiano 77, 38123 Trento, Italy; 2 Center for Sensors and Devices, Fondazione Bruno Kessler, Via Sommarive 18, 38123 Trento, Italy; 3 Dipartimento di Matematica e Informatica “Ulisse Dini”, Università degli Studi di Firenze, Viale Morgagni 67/a, 50134 Firenze, Italy

**Keywords:** MEMS, tuning fork gyroscope, mechanical sensitivity, amplification mechanism

## Abstract

This paper describes a novel micro-electro-mechanical system (MEMS) tuning fork gyroscope (TFG) design that employs a chevron-shaped displacement mechanism to amplify the displacement generated by the Coriolis force, thereby increasing the TFG’s mechanical sensitivity. This approach was evaluated using both theoretical modeling and finite element analysis (FEA), and the results showed a high degree of agreement between the two methods. A conventional TFG having a comparable area was also designed and analyzed for comparison purposes. By introducing the displacement amplification mechanism, the proposed MEMS TFG design provides an output displacement about 2.5 times higher than the conventional design, according to the computation, without increasing the device footprint. Theoretical analysis and FEA on the TFG with amplification and a conventional TFG confirmed that the amplified displacement significantly improves the mechanical sensitivity of the gyroscope compared to conventional TFG designs.

## 1. Introduction

Micro-electro-mechanical system (MEMS) gyroscopes have a wide range of applications in inertial navigation, attitude reference, and other fields due to their advantages of small size, light weight, low cost, low power consumption, and mass production [1,2,3,4]. Various types of gyroscopes were developed based on different operating principles, such as thermal gyroscopes [5,6,7], ring gyroscopes [8,9], gas rate gyroscopes [10], and vibratory gyroscopes [11,12,13]. Thermal gyroscopes operate by detecting the changes in temperature distribution caused by Coriolis forces acting on a gas flow based on thermal expansion. Ring gyroscopes utilize the interference of light traveling in opposite directions around a circular path to measure angular velocity. Gas rate gyroscopes measure angular velocity by transducing the deflection of a gas flow due to Coriolis forces. MEMS vibratory gyroscopes measure angular velocity by transducing the displacement of a Coriolis mass in the sensing axis. This can be achieved by various sensing strategies, including optical sensing, piezoelectric sensing, and capacitive sensing [11,14,15,16]. Among the different gyroscopes reported in the literature, capacitive micro mechanical gyroscopes based on electrostatic driving and capacitance detection are currently the mainstream of both research and industry. For capacitive MEMS gyroscopes, the dual-mass tuning fork gyroscope (TFG) structure is widely adopted due to its advantages of resistance to environmental influences, such as mechanical shocks and thermal fluctuations [17,18] and effective suppression of common-mode interference [12,19,20]. TFG design typically consists of two masses vibrating in opposite directions. When subjected to angular motion, the two masses are affected by Coriolis forces, causing displacements perpendicular to the vibration direction, which are then sensed and converted into angular velocity. Common-mode interference, arising from the vibrations of the two masses, is suppressed through a proper mechanical design and eliminated via differential detection [13,21]. However, the small size characteristic of the TFG limits the Coriolis mass [22,23,24], resulting in a relatively small Coriolis force and displacement caused by this force, which in turn limits the sensitivity and resolution of the sensor.

One way to enhance mechanical sensitivity is to increase the maximum motion displacement in the driving mode. However, current fabricating process conditions limit feature sizes such as comb finger gap and overlap length, making it difficult to increase the maximum motion displacement of the comb finger actuator [25]. In addition, due to the spring design, excessive driving displacement will increase the coupling between the driving and sensing modes, thereby introducing unwanted coupling between the drive and sense vibrations [26]. Consequently, alternative methods are needed to enhance the mechanical sensitivity of MEMS gyroscopes.

In recent years, mechanical amplification mechanisms have been applied in micro inertial sensors. Zeimpekis et al. [27] proposed a lever-based amplification mechanism for a capacitive micro accelerometer, which increases the change in capacitance of a comb-like capacitor on the micro accelerometer, thereby improving its sensitivity. Davies et al. [28] used a chevron displacement amplifier to amplify the displacement of the accelerometer proof mass. In this configuration, the acceleration was obtained by measuring the amplified displacement through optical interference, demonstrating the feasibility of using a chevron displacement amplifier for accelerometers. Zhang et al. [29] designed a three-degree-of-freedom micro gyroscope with an anchored lever mechanism and analyzed its gain performance. The study showed that introducing the lever can effectively improve gyroscope gain. Li et al. [30] incorporated anchored levers into the sensing mechanism of the tuning fork gyroscope (TFG), established a theoretical model, and conducted finite element analysis (FEA) to verify the feasibility of amplifying the displacement of the TFG with the anchored lever mechanism. Zhou et al. [31] introduced a silicon MEMS quad mass gyroscope (QMG) based on a diamond-shaped flexible mechanical amplification mechanism, which amplifies Coriolis displacements using two pairs of chevron displacement amplifiers and achieves a higher signal-to-noise ratio compared to a conventional QMG. However, existing research on mechanical amplification mechanisms in MEMS gyroscopes is limited and mostly based on anchored levers, with no reported studies, to the authors’ knowledge, on the application of chevron displacement amplifiers in MEMS TFGs.

This paper proposes a MEMS TFG incorporating a chevron displacement amplification mechanism. The purpose is to enhance gyroscope performance by amplifying the displacement induced by Coriolis forces, thereby increasing the sensitivity to angular motion. The proposed design is verified by building a theoretical model and using FEA simulation to evaluate its mechanical sensitivity. A MEMS TFG without an amplification mechanism is also designed, having identical proof mass and springs and a comparable footprint, to compare it with the proposed design. The rest of this paper is arranged as follows: Section 2 introduces the structure and working principle of MEMS TFG. In Section 3, the proposed MEMS TFG is theoretically analyzed according to the mechanical vibrations theory, and a mathematical model is then obtained. Section 4 provides the FEA of the proposed design to verify the effectiveness of the analysis and design. Section 5 provides the simulation results and analyzes and discusses the performance improvement achieved by the amplification structure. Finally, Section 6 concludes the paper with a summary of the findings.

## 2. Architecture Design and Working Principle

**Figure 1:**
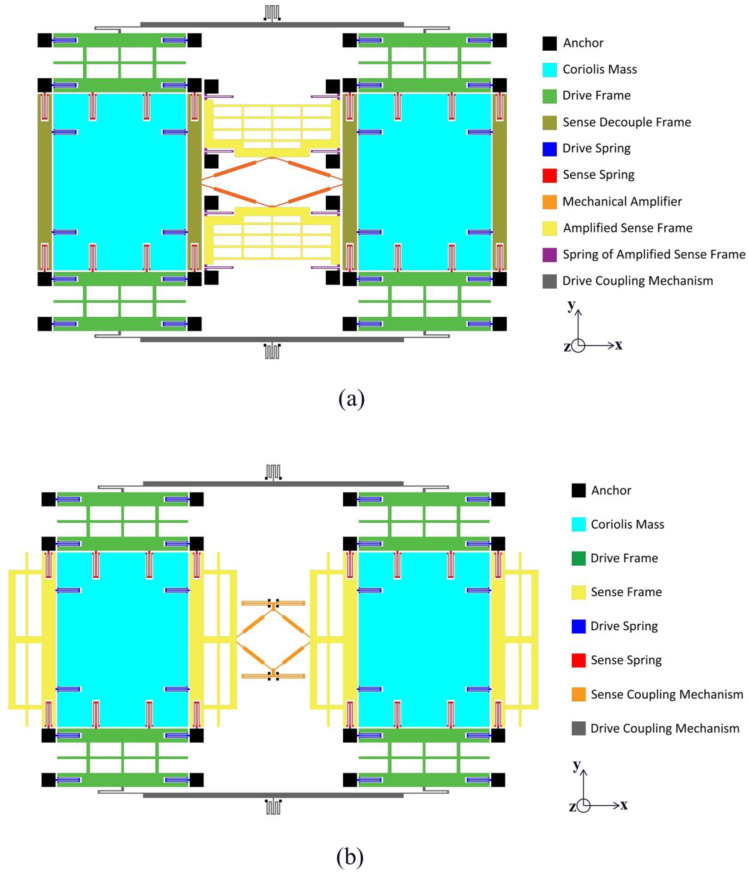
Schematic diagrams of the proposed designs of tuning fork gyroscope (TFG): (**a**) Scheme I, (**b**) Scheme II.

The MEMS gyroscope structure with the chevron displacement amplification mechanism proposed in this paper is shown in Figure 1a. The vertical direction is the driving direction, while the horizontal direction is the sensing direction. The structure implements a tuning fork configuration composed of two identical left and right tines, a pair of symmetrical chevron displacement amplification mechanisms, and two sensing frames. The sensing frames are connected to the amplifier and are supported by springs at four corners. The left and right tines are connected by a pair of long levers controlling the driving mode (i.e., the drive coupling mechanism in Figure 1a). Each tine comprises a Coriolis mass, two drive frames, two sense decoupling frames, and several springs. All springs are designed as folded flexures to ensure good linearity, allowing the micro gyroscope to work within the linear elastic deformation range of the springs. The left and right tines adopt a fully decoupled design, where the drive frames are only able to move along the vertical direction, and the sense decouple frames are only able to move along the horizontal direction to prevent interference from the drive mode to the sensing mode. For comparison purposes, a conventional MEMS TFG is designed using the same design principles, as shown in Figure 1b. The design of the two tines is the same as that of the amplified TFG, but the difference is that the sensing decoupling frame is directly used as the sensing frame. The sensing modes of the left and right tines are coupled through the diamond structure with anchored springs. The small comb electrodes on the driving and sensing frames are omitted for simplicity. Hereafter, the two designs are called Scheme I and II, respectively. Both TFG designs have comparable footprints and are symmetric along both the left–right and top–bottom axes.

When the gyroscope operates by controlling the electrostatic driving force, the driving structure of the two tines drives the Coriolis mass to vibrate harmonically in an anti-phase mode along the driving axis in the vertical direction. When the gyroscope rotates at an angular velocity directed outside its plane, the left and right proof masses will be affected by opposite Coriolis forces along the sensing axis, generating opposite phase vibrations of the two sensing masses along the sensing axis, which cause the anti-phase vibrations of the sensing decoupling frames. The angular velocity is obtained by measuring the amplitude of the sensing mode vibrations. For Scheme I, the vibrations of the left and right sensing decoupling frames are amplified by chevron displacement amplifiers and then measured by the electrodes on the sensing frame after displacement amplification. In Scheme II, since the sensing decoupling frames are directly used as the sensing frames, the vibrations of the left and right sensing decoupling frames are directly measured by the electrodes on them.

## 3. Theoretical Analysis of the Proposed TFGs

For a MEMS vibratory gyroscope, the vibration characteristics of its mechanical structure, which are the natural frequency and mode shape, are one of the primary considerations in the design. To assess the gyroscope performance, the frequency response of vibration is needed to compute the mechanical sensitivity of the gyroscope. In this section, the theoretical analysis of the two designed MEMS TFGs is carried out.

### 3.1. Analysis of Scheme I

**Figure 2:**
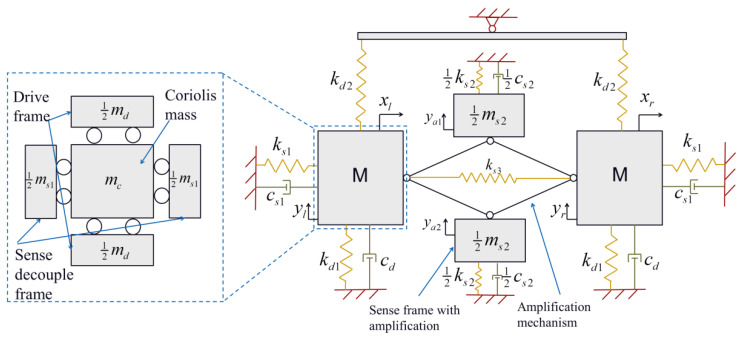
Schematic diagram of the lumped parameter model of Scheme I.

The schematic diagram of the lumped parameter model of the Scheme I gyroscope is shown in Figure 2. Since the mass of the folded beam spring is negligible relative to the mass of the driving frame, the sensing frame, and the Coriolis mass, the springs are treated as massless. Furthermore, because the mass of the beams of the amplification mechanism is far smaller than that of the frames and Coriolis masses, they are regarded as massless linkages.

#### 3.1.1. Amplification Mechanism Analysis

**Figure 3:**
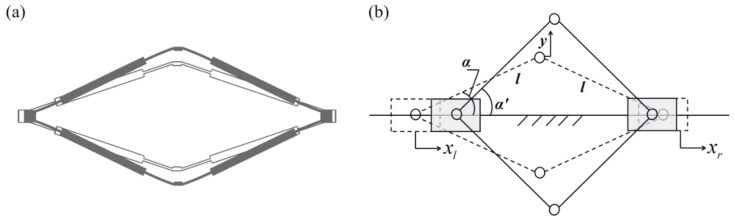
Displacement amplification mechanism: (**a**) overall structure, (**b**) kinematic model schematic diagram.

The displacement amplification structure is the key component of the proposed gyroscope structure. This comprises a pair of chevron-shaped displacement amplifiers symmetrically positioned above and below the central horizontal axis, as shown in Figure 3a. The kinematic model of the structure is shown in Figure 3b.

As is shown in Figure 3b, when the left and right ends of the amplifier move along the horizontal axis with displacements $x_{l}$ and $x_{r}$, respectively, according to the law of trigonometry, the horizontal inclination angle of each arm of the amplifier can be represented as

(1) $$\alpha^{\prime }=\cos^{-1}\left(\frac{2l\cos\alpha -\left(x_{l}-x_{r}\right)}{2l}\right),$$

where α is the horizontal inclination angle of each arm at rest position and l is the length of each arm.

Therefore, the vertical displacement of the amplified end is

(2) $$y=l\sin\alpha^{\prime }-l\sin\alpha =\sqrt{l^{2}-{\left(l\cos\alpha -\frac{1}{2}\left(x_{l}-x_{r}\right)\right)}^{2}}-l\sin\alpha .$$

Since the displacement caused by Coriolis force, which is the displacement input at the left and right ends of the amplifier, is relatively small compared to the amplifier arm length, a small displacement assumption can be made. Using a first-order Taylor polynomial to simplify Equation (2), the vertical displacement becomes

(3) $$y=\frac{1}{2}\cot\alpha \left(x_{l}-x_{r}\right).$$

#### 3.1.2. Dynamics and Sensitivity Analysis

During operation, the left and right tines are driven by a pair of harmonic excitation forces with opposite phases along the drive axis, denoted as $F_{d}\sin(\omega t)$ and $-F_{d}\sin(\omega t)$ with the operation frequency as ω. The equations of motion of each tine at drive mode are

(4) $$\left(m_{d}+m_{c}\right){\ddot{y}}_{l}+c_{d}{\dot{y}}_{l}+\left(k_{d1}+k_{d2}\right)y_{l}=F_{d}\sin(\omega t),$$

(5) $$\left(m_{d}+m_{c}\right){\ddot{y}}_{r}+c_{d}{\dot{y}}_{r}+\left(k_{d1}+k_{d2}\right)y_{r}=-F_{d}\sin(\omega t).$$

where $y_{l}$ and $y_{r}$ are the displacement of left and right Coriolis mass in the drive direction, $m_{d}$ and $m_{c}$ represent the mass of drive frames and the Coriolis mass, $k_{d1}$ and $k_{d2}$ are the stiffness of drive springs and drive couple springs, and $c_{d}$ is the damping coefficient of each tine in drive mode. From Equations (4) and (5), the drive velocities of each tine at resonance can be obtained, which are

(6) $${\dot{y}}_{l}=\frac{F_{d}}{c_{d}}\sin(\omega_{nd}t),\;\;\;\;\;{\dot{y}}_{r}=-\frac{F_{d}}{c_{d}}\sin(\omega_{nd}t),$$

in which $\omega_{nd}=\sqrt{\frac{k_{d1}+k_{d2}}{m_{d}+m_{c}}}$ is the natural frequency at drive mode.

The equations of motion at sense mode can be computed by Euler–Lagrange equations,

(7) $$\frac{d}{dt}\left(\frac{\partial L}{\partial {\dot{x}}_{l}}\right)-\frac{\partial L}{\partial x_{l}}=-2m_{c}\Omega_{z}{\dot{y}}_{l}-\frac{\partial D}{\partial {\dot{x}}_{l}},$$

(8) $$\frac{d}{dt}\left(\frac{\partial L}{\partial {\dot{x}}_{r}}\right)-\frac{\partial L}{\partial x_{r}}=-2m_{c}\Omega_{z}{\dot{y}}_{r}-\frac{\partial D}{\partial {\dot{x}}_{r}},$$

where $x_{l}$ and $x_{r}$ are the displacements of left and right Coriolis mass in the sense direction, L=T−V is the Lagrangian function of the system in which *T* and *V* are the kinetic energy and potential energy, *D* is Rayleigh dissipation function, and $-2m_{c}\Omega_{z}{\dot{y}}_{l}$ and $-2m_{c}\Omega_{z}{\dot{y}}_{r}$ are the Coriolis forces exerted on the left and right proof masses with $\Omega_{z}$ as the angular rate that the gyroscope experiences.

In the sense mode operation, the Coriolis mass and sense decoupling frames of the left and right tines vibrate along the horizontal axis with amplitude $x_{l}$ and $x_{r}$, which induces vibrations of the two amplified sense frames along the vertical axis with magnified amplitude $y_{a1}$ and $y_{a2}$. Based on Equation (3), assuming the amplification ratio of the amplifier as $R=\frac{1}{2}\cot\alpha$, the vibration amplitudes become

(9) $$y_{a1}=R\left(x_{l}-x_{r}\right),\;\;\;\;\;y_{a2}=-R\left(x_{l}-x_{r}\right).$$

The kinetic energy *T*, potential energy *V*, and dissipation function *D* can be written as

(10) $$T=\frac{1}{2}\left(m_{s1}+m_{c}\right){\dot{x}}_{l}^{2}+\frac{1}{4}m_{s2}{\dot{y}}_{a1}^{2}+\frac{1}{4}m_{s2}{\dot{y}}_{a2}^{2}+\frac{1}{2}\left(m_{s1}+m_{c}\right){\dot{x}}_{r}^{2},$$

(11) $$V=\frac{1}{2}k_{s1}x_{l}^{2}+\frac{1}{4}k_{s2}y_{a1}^{2}+\frac{1}{4}k_{s2}y_{a2}^{2}+\frac{1}{2}k_{s1}x_{r}^{2}+\frac{1}{2}k_{s3}{\left(x_{l}-x_{r}\right)}^{2},$$

(12) $$D=\frac{1}{2}c_{s1}{\dot{x}}_{l}^{2}+\frac{1}{4}c_{s2}{\dot{y}}_{a1}^{2}+\frac{1}{4}c_{s2}{\dot{y}}_{a2}^{2}+\frac{1}{2}c_{s1}{\dot{x}}_{r}^{2}.$$

where $m_{s1}$ is the mass of sense decouple frames for each tine, $m_{s2}$ is the mass of the amplified sense frames, $k_{s1}$ is the stiffness of sense springs on each tine, $k_{s2}$ is the stiffness of the springs on the amplified sense frames, $c_{s1}$ is the damping coefficient corresponding to the proof mass on each tine, and $c_{s2}$ is the damping coefficient corresponding to the amplified sense frames.

Combining Equations (7)–(12), the equations of motion are derived as

(13) $$R^{2}\left(m_{s2}({\ddot{x}}_{l}-{\ddot{x}}_{r})+c_{s2}({\dot{x}}_{l}-{\dot{x}}_{r})+k_{s2}(x_{l}-x_{r})\right)+(m_{c}+m_{s1}){\ddot{x}}_{l}+c_{s1}{\dot{x}}_{l}+k_{s1}x_{l}+k_{s3}(x_{l}-x_{r})=-2m_{c}\Omega_{z}{\dot{y}}_{l},$$

(14) $$-R^{2}\left(m_{s2}({\ddot{x}}_{l}-{\ddot{x}}_{r})+c_{s2}({\dot{x}}_{l}-{\dot{x}}_{r})+k_{s2}(x_{l}-x_{r})\right)+(m_{c}+m_{s1}){\ddot{x}}_{r}+c_{s1}{\dot{x}}_{r}+k_{s1}x_{r}-k_{s3}(x_{l}-x_{r})=-2m_{c}\Omega_{z}{\dot{y}}_{r}.$$

As the sense mode is based on the anti-phase vibration of the two tines, the anti-phase and in-phase displacements $x_{an}$ and $x_{in}$ are introduced as follows:

(15) $$x_{\mathrm{an}}=x_{l}-x_{r},\;\;\;\;\;x_{\mathrm{in}}=x_{l}+x_{r}.$$

Combining Equations (13)–(15), the equations of motion with respect to in-phase and anti-phase motions are obtained as

(16) $$\left(m_{s1}+m_{c}\right){\ddot{x}}_{in}+c_{s1}{\dot{x}}_{in}+k_{s1}x_{in}=0,$$

(17) $$\left(m_{c}+m_{s1}+2R^{2}m_{s2}\right){\ddot{x}}_{an}+\left(c_{s1}+2R^{2}c_{s2}\right){\dot{x}}_{an}+\left(k_{s1}+2R^{2}k_{s2}+2k_{s3}\right)x_{an}=2F_{c}\sin(\omega_{nd}t),$$

where $F_{c}$ is the magnitude of the Coriolis force, represented as

(18) $$F_{c}=-2m_{c}\Omega_{z}\frac{F_{d}}{c_{d}}.$$

Rewriting Equations (16) and (17) into matrix from, the following equation results as follows:

(19) $$M\ddot{x}+C\dot{x}+Kx=F_{c}e^{i\omega t},$$

where $M=\left(\begin{array}{cc} m_{s1}+m_{c} & 0\\ 0 & m_{c}+m_{s1}+2R^{2}m_{s2} \end{array}\right)$, $C=\left(\begin{array}{cc} c_{s1} & 0\\ 0 & 2R^{2}c_{s2}+c_{s1} \end{array}\right)$, $K=\left(\begin{array}{cc} k_{s1} & 0\\ 0 & k_{s1}+2R^{2}k_{s2}+2k_{s3} \end{array}\right)$, $F_{c}=\left(\begin{array}{c} 0\\ 2F_{c} \end{array}\right)$, and $x=\left(\begin{array}{c} x_{in}\\ x_{an} \end{array}\right)$.

Therefore, the natural frequencies of the sense mode can be obtained by solving the characteristic equation $\left|K-\omega^{2}M\right|=0$, which yields

(20) $$\omega_{an}^{2}=\frac{k_{s1}+2R^{2}k_{s2}+2k_{s3}}{2R^{2}m_{s2}+m_{c}+m_{s1}},\;\;\;\;\;\omega_{in}^{2}=\frac{k_{s1}}{m_{c}+m_{s1}},$$

where $\omega_{an}$ and $\omega_{in}$ are the anti-phase and in-phase natural frequencies of the sense mode. It is also noticeable that the value of $k_{s1}$ is different in the above expressions because the mechanical amplifier has different spring stiffnesses corresponding to in-phase and anti-phase vibrations. Therefore, the anti-phase mode for readout is well separated from the in-phase mode.

The frequency response can be computed by substituting the steady-state solution $x\;=\;Xe^{i\omega t}$ into Equation (19), and the amplitude matrix can be computed as

(21) $$X={\left(K-\omega^{2}M+i\omega C\right)}^{-1}F_{c}.$$

Hence, the steady-state amplitude in sense mode can be written as

(22) $$x_{an}=\left|\frac{2F_{c}}{k_{an}-\omega^{2}m_{an}+i\omega c_{an}}\right|=\frac{2\left|F_{c}\right|}{\sqrt{{\left(k_{an}-\omega^{2}m_{an}\right)}^{2}+{\left(\omega c_{an}\right)}^{2}}},$$

where $k_{an}=k_{s1}+2R^{2}k_{s2}+2k_{s3}$, $m_{an}=2R^{2}m_{s2}+m_{c}+m_{s1}$, and $c_{an}=2R^{2}c_{s2}+c_{s1}$.

Therefore, when the gyroscope has a matching mode of drive and sense frequency, the mechanical sensitivity of Scheme I can be represented as

(23) $$S_{I}=\frac{y_{a1}-y_{a2}}{\Omega_{z}}=\frac{8Rm_{c}F_{d}}{c_{d}\sqrt{{\left(k_{an}-\omega^{2}m_{an}\right)}^{2}+{\left(\omega c_{an}\right)}^{2}}}.$$

### 3.2. Analysis of Scheme II

**Figure 4:**
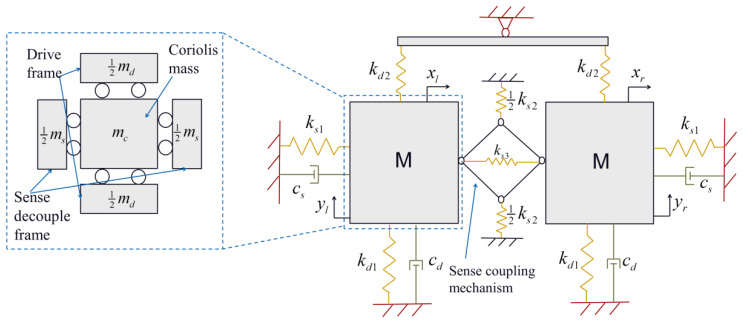
Schematic diagram of the lumped parameter model of Scheme II.

For the design of Scheme II, the same simplifications as the Scheme I model are adopted to obtain the schematic diagram of the lumped parameter model, as shown in Figure 4.

The drive mode of Scheme II is the same as Scheme I. Adopting the same approach as the analysis of Scheme I at sense mode, there is

(24) $$M^{\prime }\ddot{x}+C^{\prime }\dot{x}+K^{\prime }x=F_{c}e^{i\omega t},$$

in which $M^{\prime }=\left(\begin{array}{cc} m_{c}+m_{s} & 0\\ 0 & m_{c}+m_{s} \end{array}\right)$, $K^{\prime }=\left(\begin{array}{cc} k_{s1} & 0\\ 0 & k_{s1}+2R^{2}k_{s2}+2k_{s3} \end{array}\right)$ and $C^{\prime }=\left(\begin{array}{cc} c_{s} & 0\\ 0 & c_{s} \end{array}\right)$.

Natural frequencies at sense mode are

(25) $${\omega^{\prime }}_{an}^{2}=\frac{k_{s1}+2R^{2}k_{s2}+2k_{s3}}{m_{c}+m_{s}},\;\;\;\;\;{\omega^{\prime }}_{in}^{2}=\frac{k_{s1}}{m_{c}+m_{s}},$$

in which $\omega_{an}^{\prime }$ and $\omega_{in}^{\prime }$ are anti-phase and in-phase natural frequencies with various values of $k_{s1}$ due to the sense coupling mechanism structure having different spring stiffnesses corresponding to in-phase and anti-phase vibrations.

Assuming $k_{an}^{\prime }=k_{s1}+2R^{2}k_{s2}+2k_{s3}$ and $m_{an}^{\prime }=m_{c}+m_{s}$, the steady-state amplitude and total mechanical sensitivity can be derived as

(26) $$x_{an}^{\prime }=\frac{2\left|F_{c}\right|}{\sqrt{{\left(k_{an}-\omega^{2}m_{an}^{\prime }\right)}^{2}+{\left(\omega c_{s}\right)}^{2}}},$$

(27) $$S_{\mathrm{II}}=\frac{x_{l}-x_{r}}{\Omega_{z}}=\frac{4m_{c}F_{d}}{c_{d}\sqrt{{\left(k_{an}^{\prime }-\omega^{2}m_{an}^{\prime }\right)}^{2}+{\left(\omega c_{s}\right)}^{2}}}.$$

## 4. Finite Element Analysis of the Proposed Design

Finite element analysis (FEA) was carried out using COMSOL software (version 6.0) to investigate the dynamic behavior and structural performance of the proposed TFGs and to verify the effectiveness of the theoretical analysis. The design parameters of the TFGs being analyzed are listed in Table 1.

**Figure 5:**
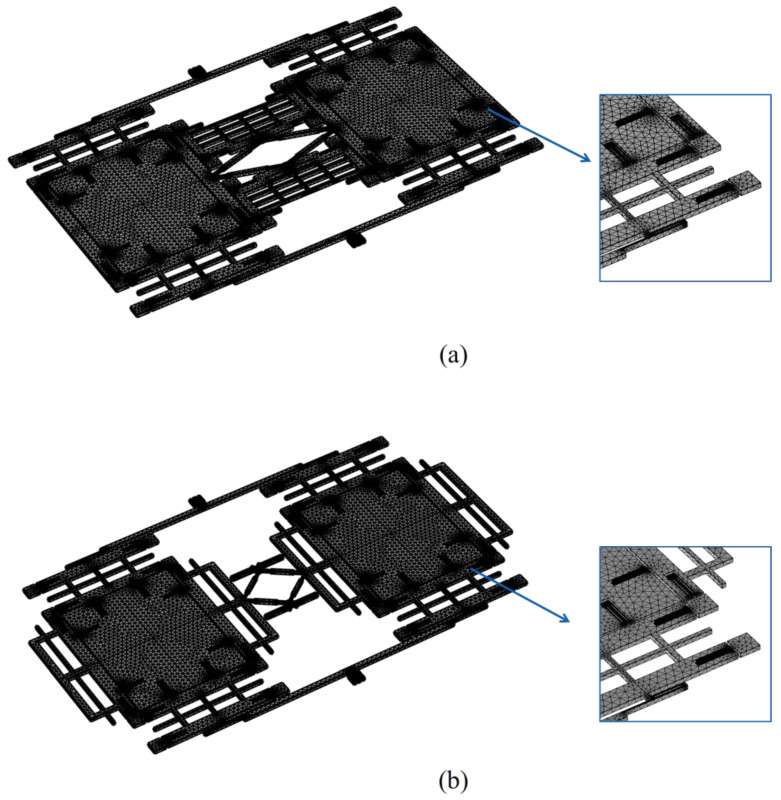
Finite element meshing: (**a**) Scheme I, (**b**) Scheme II.

The 3D geometry of the TFG was built and the material was assigned as silicon, with the properties shown in Table 2. The boundary conditions were set to simulate the attachment of the gyroscope to a substrate, with fixed constraints at the anchor points and loads applied to simulate Coriolis forces during operation. The mesh elements of tetrahedrons were built with a physics-controlled meshing strategy, and the element for spring flexures was treated with extra refining to ensure the accuracy of the FEA results. The meshing of Schemes I and II are shown in Figure 5a,b, respectively. All mesh elements are three-dimensional tetrahedrons with second-order discretization. The element quantities of the meshed models are 410,550 for Scheme I and 408,672 for Scheme II.

Modal analysis was carried out to identify the natural frequency and mode shape of the proposed TFGs. Harmonic analyses were also carried out to investigate the dynamic behavior and evaluate the mechanical sensitivity of the designs of TFGs.

## 5. Results and Discussion

### 5.1. Static Analysis

Static structural finite element analysis is used to obtain the stiffness of the proposed TFG structures and to verify the kinematics of the proposed amplification mechanism.

**Figure 6:**
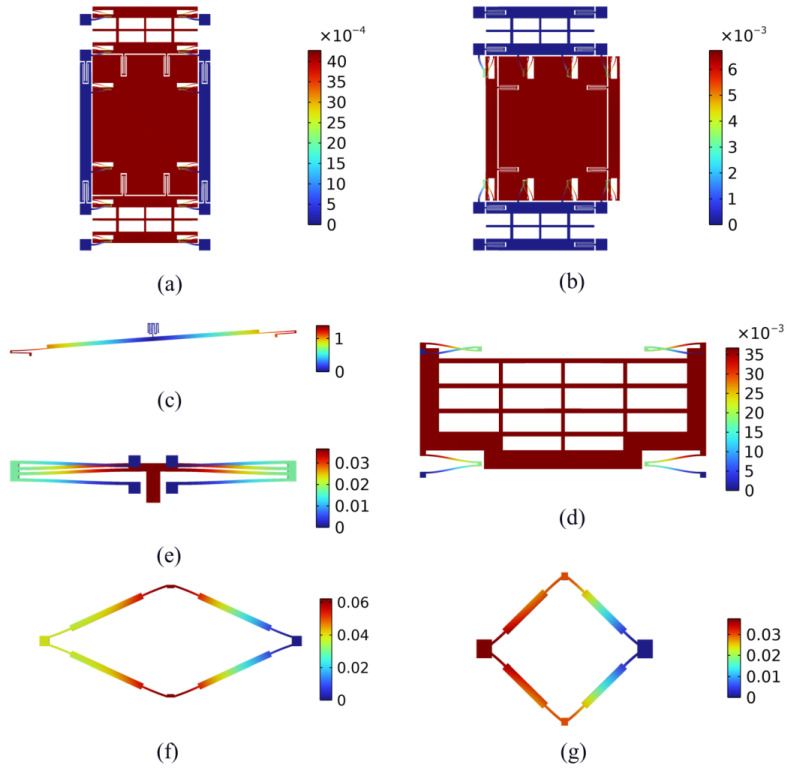
Deformations of the proposed TFGs (color-bar units: μm): (**a**) drive springs, (**b**) sense springs, (**c**) drive couple lever, (**d**) amplified sense frame of Scheme I, (**e**) sense couple spring of Scheme II, (**f**) amplification mechanism of Scheme I, (**g**) sense couple mechanism of Scheme II.

The stiffness values corresponding to the drive and sense modes of the two proposed TFG structures are analyzed. The deformations of the structures under a 10 μN force are computed to calculate the stiffness values, as shown in Figure 6. Stiffness values can be calculated as the ratio of force to deformation based on the obtained deformation values. The calculated stiffness values are shown in Table 3.

**Figure 7:**
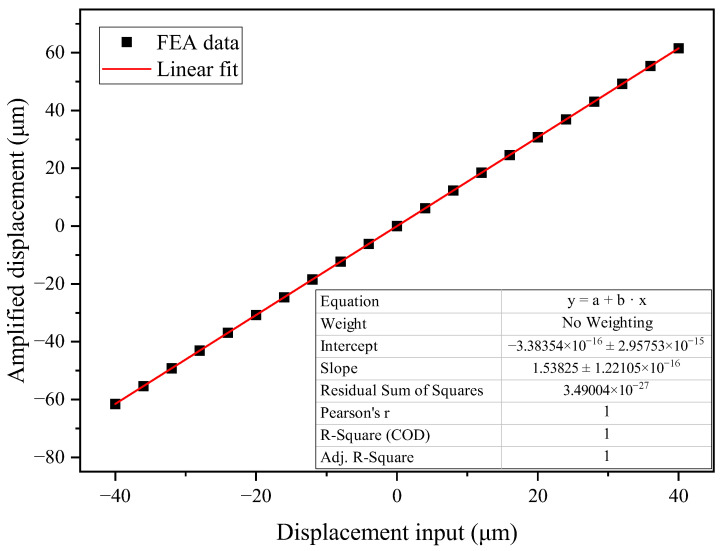
Simulated amplified displacement and linear fit.

The kinematics of the chevron-shape amplification mechanism is analyzed based on FEA. The amplified displacements with respect to different displacement inputs are computed. The results are shown in Figure 7, where the displacement input represents the differential displacement between the left and right ends of the amplifier.

As depicted in Figure 7, the amplified displacement and displacement input provide a good linearity. The calculated slope is 1.53825, close to the theoretical value of 1.53884 calculated according to Equation (3), with a difference of 0.038%. Therefore, the amplification mechanism provides a linear output within the displacement range of the TFG’s operation. Meanwhile, a close agreement between FEA results and theoretical analysis of the amplification mechanism is obtained, validating the accuracy of the theoretical analysis for the proposed amplification mechanism.

### 5.2. Modal Analysis

**Figure 8:**
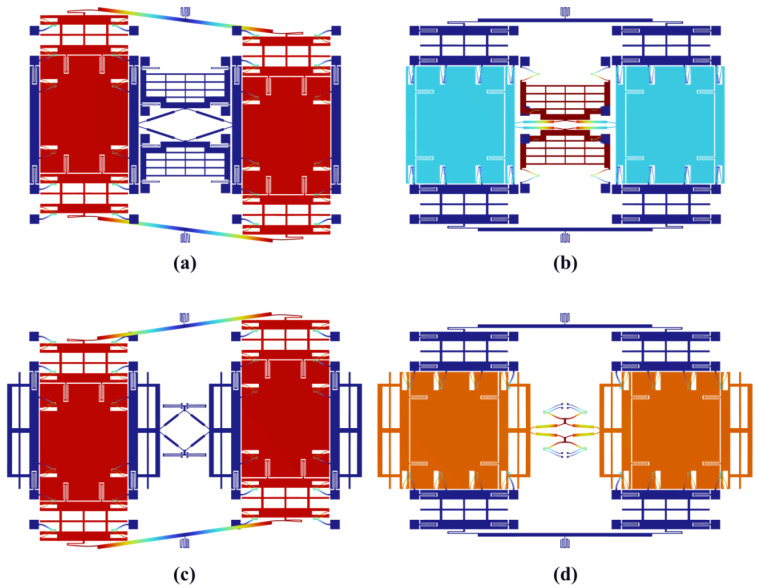
Mode shape obtained by FEA: (**a**) Scheme I drive mode, (**b**) Scheme I sense mode, (**c**) Scheme II drive mode, (**d**) Scheme II sense mode.

The modal analysis results are crucial for investigating the dynamic behavior of the MEMS TFGs, especially for the Coriolis response detection mode and anti-phase driving mode, which operate near resonances. The drive and sense modes of Schemes I and II are shown in Figure 8. In addition, higher-order modes such as torsional or out-of-plane vibrations are identified, with frequencies significantly higher than the operating range, ensuring minimal interference with the primary drive and sense modes. The modal shape confirms that the gyroscope maintains in-plane motion at the operating frequency, which is crucial for accurate angular velocity detection.

The natural frequencies corresponding to Scheme I and II obtained through FEA and modal analysis on drive and sense modes in the theoretical model are listed in Table 4, showing a good agreement between the analytical and finite element models.

### 5.3. Frequency Response and Sensitivity Analysis

Frequency response analysis is used to understand the gyroscope’s vibrational output response and to evaluate the mechanical sensitivity. The frequency response of MEMS TFGs is analyzed to obtain their vibrational amplitudes under different vibration frequencies. The response of the device to harmonic excitation is analyzed, and the relationship between the vibrational displacement and input frequency is focused. In our analysis, the damping sources are considered viscous air damping on drive and sense frame electrodes, and damping on the proof masses. The damping coefficient values are calculated using the methods given in [32,33]. For a fair comparison, the damping coefficients of the sense frame electrodes of Schemes I and II are based on the same number of electrodes. The values used for calculation and the damping coefficient values are shown in Table 5.

**Figure 9:**
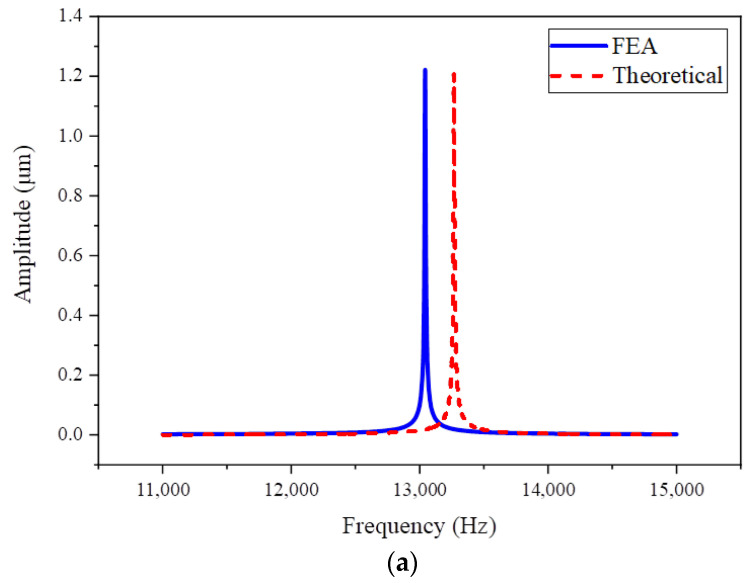

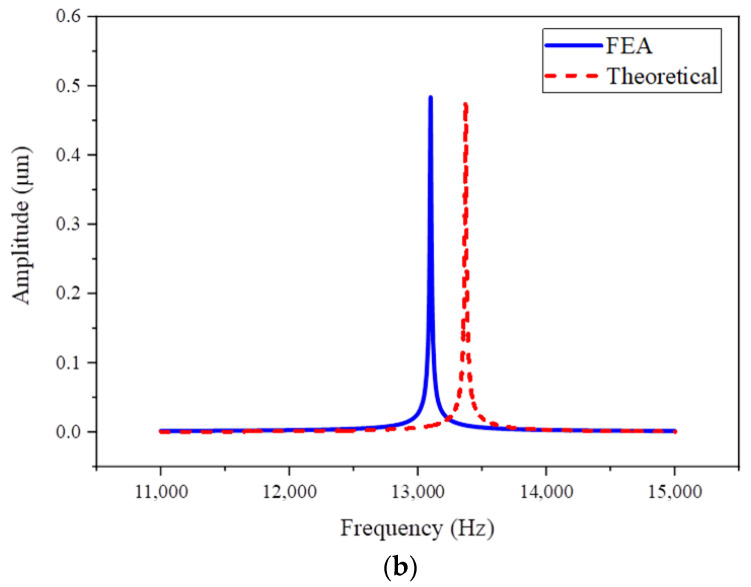
Frequency response plots of investigated TFGs: (**a**) Scheme I, (**b**) Scheme II.

Based on the modal analysis results, the frequency sweep range for drive and sense modes is determined, which is set from 11,000 Hz to 15,000 Hz. The step is set to 0.1 Hz in an interval of 20 Hz near the resonance frequency, 1 Hz in the rest of the interval of 100 Hz near the resonance frequency, and 10 Hz for the rest to obtain more resolution in the concerned frequency range. The frequency response is then calculated by applying a pair of anti-phase harmonic excitation forces with a magnitude of 1 μN along the sense axis. The frequency response plots are shown in Figure 9, where the amplitudes correspond to the vibrations of the amplified sense frame of Scheme I and the sense frame of Scheme II.

Based on the frequency response, the quality factors Q are obtained, with the values of 1069.11 and 1052.79 for FEA and the theoretical model in Scheme I, and 700.57 and 692.78 for FEA and the theoretical model in Scheme II. It can be seen from the frequency responses that the theoretical analysis and FEA results provide a consistent agreement with a slight difference of less than 2% in terms of natural frequency and amplitude. The vibrational amplitudes of Scheme I and II at resonance are reported in Table 6.

As reported in Table 6, it can be seen that the proposed design (Scheme I) achieves a larger resonance amplitude value on the sense components compared with Scheme II. Compared with the conventional design, the proposed design achieves a resonance amplitude output ~2.5 times higher under the same excitation input. Therefore, it can be concluded that the proposed design of the TFG with a mechanical amplification structure provides a higher mechanical sensitivity than the conventional design.

### 5.4. Effectiveness and Validity of the Models

**Figure 10:**
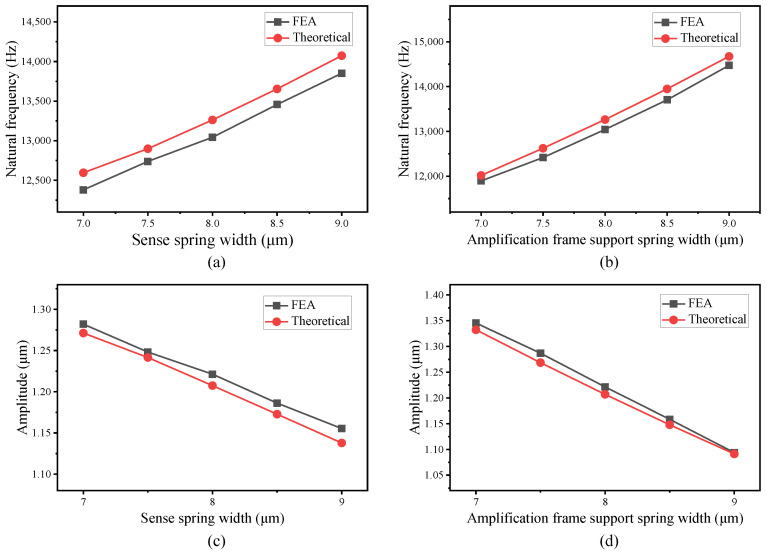
Sense mode natural frequency with varying sense spring width (**a**) and amplification frame support spring width (**b**); resonant amplitude of the sense frame with varying sense spring width (**c**) and amplification frame support spring width (**d**) for analytical model and FEA of Scheme I.

**Figure 11:**
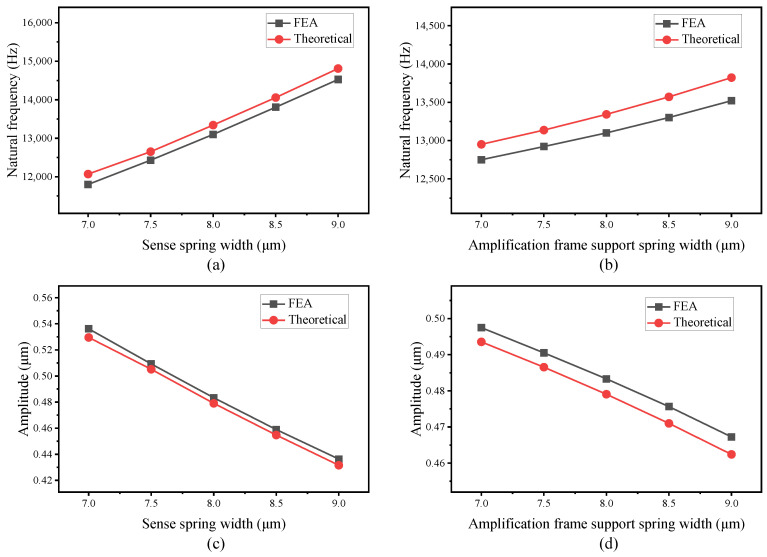
Sense mode natural frequency with varying sense spring width (**a**) and amplification frame support spring width (**b**); resonant amplitude of the sense frame with varying sense spring width (**c**) and amplification frame support spring width (**d**) for analytical model and FEA of Scheme II.

The static analysis, modal analysis, and harmonic response results from the theoretical model and FEA of both designs are obtained, showing a close agreement between the analytical model and FEA. To further investigate the validity and effectiveness of the analytical and finite element models, simulations are carried out by varying the size of different design parameters in a technologically relevant interval. The design parameters chosen to explore the effectiveness of the models are the width of the sense spring, the width of the amplification frame support spring in Scheme I, and the width of the sense coupling support spring in Scheme II. The natural frequencies and resonant amplitudes are computed with both the theoretical model and FEA under different values of the above design parameters. The results obtained and the difference between theoretical analysis and FEA are plotted in Figure 10 and Figure 11 for Scheme I and Scheme II, respectively.

As is shown in Figure 10 and Figure 11, the values obtained by theoretical analysis are consistent with the results provided by FEA with a variety of different design parameters, which further proves the effectiveness of the theoretical model. It can also be seen that the differences between the theoretical model and FEA are within a reasonable range, with a maximum difference of 1.76% for natural frequency and 1.54% for amplitude in Scheme I, and 2.22% for natural frequency and 1.25% for amplitude in Scheme II. Such deviation can be ascribed to the assumption made in the theoretical analysis. For example, the theoretical model neglected the mass of the amplification structure chevron shape beams because that is a much smaller mass than the other structures. On the other hand, the close agreement of the results between theoretical analysis and FEA proves that these assumptions and simplifications are reasonable.

### 5.5. Discussion

As can be seen from the results obtained from FEA and theoretical analysis, the natural frequencies of the drive and sense modes show a slight mismatch in both the proposed Schemes I and II, depicted in Table 4. To achieve a larger mechanical sensitivity of the gyroscope, the natural frequencies should be fine-tuned by changing the spring’s stiffness to eliminate the mismatch. However, due to the existence of fabrication tolerances, the mismatch normally requires natural frequency tuning by applying an extra electrostatic force to change the gyroscope’s stiffness along drive or sense direction.

Furthermore, it is noticeable that adding amplification mechanisms also increases the complexity of fabrication, which requires higher accuracy for maintaining uniformity in the springs and good alignments between the two pairs of chevron-shaped beams. Meanwhile, the trade-off between displacement amplification and damping should be considered. Although the amplification structure amplifies the Coriolis displacement, it also introduces additional damping, which decreases the Coriolis displacement on the Coriolis mass. However, this reduction is more than compensated by the larger displacement resulting from the amplification mechanism. For specific designs, this trade-off should be considered and it is best to optimize the amplification ratio to obtain the optimal sensitivity.

## 6. Conclusions

This study reported the design, analysis, and simulation of a MEMS TFG enriched with a mechanical amplification structure. By integrating mechanical amplification, the proposed design addressed the inherent sensitivity limitations of conventional TFG designs. The performance of the proposed gyroscope was evaluated by building theoretical models and carrying out finite element analysis. By comparing the proposed TFG design with a conventional TFG design, the results confirm that the amplification structure can indeed increase the mechanical sensitivity of the TFG. Meanwhile, FEA simulation results demonstrated a close agreement with theoretical analysis under variations in different design parameters, showing the validity and effectiveness of the theoretical model. Based on theoretical analysis and FEA results, it can be concluded that the proposed TFG with an amplification mechanism effectively increases the Coriolis displacements compared to a conventional TFG design, providing feasibility to enhance the sensitivity of MEMS TFGs on angular speeds transducing without impacting the device size. Future work will focus on the fabrication of the proposed TFGs on silicon-on-insulator wafers with a deep reactive ion etching technique and testing.

## Figures and Tables

**Table 1:** Design parameters of the proposed TFGs.

Parameter	Design Value
Coriolis mass volume	0.1127 mm^3^
Drive frame volume	0.0390 mm^3^
Sense frame volume of Scheme I	0.0212 mm^3^
Sense amplification frame volume	0.0320 mm^3^
Sense frame volume of Scheme II	0.0402 mm^3^
Length of the drive spring	245 μm
Length of the sense spring	250 μm
Length of amplification frame support springs	280 μm
Length of sense coupling support springs	290 μm
Width of springs	8 μm
Horizontal angle of amplification beam of Scheme I	18°
Horizontal angle of sense couple beam of Scheme II	38°
Thickness of the device layer	40 μm

**Table 2:** Material properties used in FEA.

Parameter	Value
Density	2320 kg/m^3^
Young’s modulus	170 GPa
Poisson’s ratio	0.22

**Table 3:** Stiffness values of the designed TFG structures.

Parameter	Value
Scheme I and II $k_{d1}$	2372.2 N/m
Scheme I and II $k_{d2}$	14.34 N/m
Scheme I and II $k_{s1}$	1498.1 N/m
Scheme I $k_{s2}$	544.9 N/m
Scheme II $k_{s2}$	547.7 N/m
Scheme I $k_{s3}$	259.9 N/m
Scheme II $k_{s3}$	266.7 N/m

**Table 4:** Natural frequencies of TFGs.

	Scheme I		Scheme II	
	Drive Mode	Sense Mode	Drive Mode	Sense Mode
Theoretical	13,105.9 Hz	13,265.1 Hz	13,105.9 Hz	13,342.5 Hz
FEA	12,918.7 Hz	13,043.1 Hz	12,924.7 Hz	13,100.6 Hz
Difference	1.43%	1.67%	1.38%	1.81%

**Table 5:** Parameters used in damping calculation and damping coefficients.

Parameter	Description	Value
$A_{pm}$	Area of proof mass	2.82 × 10^6^ µm^2^
$A_{se}$	Area of sense electrodes overlap	1.152 × 10^5^ µm^2^
$A_{de}$	Area of drive electrodes overlap	3.2 × 10^5^ µm^2^
$d_{pm}$	Gap between proof mass and substrate	2 µm
$d_{e}$	Gap between the electrodes	3 µm
μ	Air viscosity	1.837 × 10^−5^ Ns/m^2^
λ	Mean free path of air	0.07 µm
$c_{d}$	Damping coefficient in drive axis	2.61 × 10^−5^ Ns/m
$c_{s1}$	Damping coefficient in sense axis of Scheme I	2.42 × 10^−5^ Ns/m
$c_{s2}$	Damping coefficient of amplified frame of Scheme I	1.35 × 10^−6^ Ns/m
$c_{s}$	Damping coefficient in sense axis of Scheme II	2.49 × 10^−5^ Ns/m

**Table 6:** Sense mode vibrational amplitudes of the proposed TFGs.

	Theoretical	FEA	Difference
Scheme I	1.2072 µm	1.2214 µm	−1.193%
Scheme II	0.4790 µm	0.4833 µm	−0.898%

## Data Availability

Data are available within the article.
